# Goserelin plus endocrine treatments maintained long-term clinical benefit in a male patient with advanced breast cancer

**DOI:** 10.1186/1477-7819-12-393

**Published:** 2014-12-23

**Authors:** Hang Jiang, Tao Wang, Zefei Jiang

**Affiliations:** Department of Breast Oncology, 307 Hospital of PLA, Beijing, 100071 China

**Keywords:** Male breast cancer, Goserelin, Endocrine treatment, Family time

## Abstract

**Background:**

Goserelin plus aromatase inhibitors (AI) have already been used in male advanced breast cancer, but the cases that fulvestrantin male breast cancer are rare.

**Case presentation:**

Here we report a case of long-term (3 years) response to Goserelin plus continuing endocrine treatments given for a male advanced breast cancer. The patient prolongs his life with high life quality, and has more time with his family.

**Conclusion:**

Goserelin plus endocrine treatments may benefit male breast cancer.

## Background

Breast cancer in men is relatively rare, and most breast cancers in men are estrogen receptor/progesterone receptor positive. Tamoxifen remains the standard adjuvant treatment and is also the mainstay first-line treatment for advanced disease. However, the role of AI and fulvestrant remains controversial. We have already used goserelin plus aromatase inhibitors (AI) in male advanced breast cancer, but the cases in which fulvestrant has been used in male breast cancer are rare.

## Case presentation

A 44-year-old man was diagnosed with a left breast cancer (pT1N1M0) after left modified radical mastectomy in local hospital. The tumor was ER-positive, PgR-negative, and HER-2-negative as evaluated by immunohistochemistry. The patient received CAF(Cytoxan+doxorubicin+5-Fu) adjuvant chemotherapy with six cycles, and tamoxifen was administered for 3 years. In 2008, metastasis was found in the sternum and scapula by skeleton ECT(emission computed tomography) scan. Sternum dissection was carried out followed by radiotherapy (60 Gy total dose), and two cycles of chemotherapy with docetaxel and cisplatin were given, followed by four cycles of docetaxel and capecitabine, and monthly Zoledronic acid. Letrozole was started as the first-line endocrine treatment, but discontinued after 7 months because of disease progression.

In October 2009, the patient went to our hospital and was diagnosed with multiple bone and lung metastases on chest computed tomography (CT). He received Goserelin plus anastrozole as a second-line endocrine treatment, which resulted in stable disease for 12 months prior to progression. Goserelin plus exemestanewas started as a third-line endocrine treatment, which also resulted in stable disease for 11 months. He received 10 months of medroxyprogesterone prior to further progression of lung disease. In August 2012, Goserelin plus fulvestrant(500 mg/d,1,250 mg intramuscular monthly)was initiated as the fifth-line endocrine treatment. The lung metastasis shrank after 2 months and the patient is still on treatment. Detailed treatment information is given Table 1.

**Table 1:** **Patient’s treatment after metastasis**

Treatment line	Start and stop time, year/month	Sites of metastasis	Therapeutic schedule	Best efficacy	TTP
1st	2008/8 to 2008/10	Bone	Docetaxel + cisplatin	SD	TTP = 2 months
2nd	2008/10 to 2008/12	Bone	Docetaxel + capecitabine	SD	TTP = 4 months
3rd	2009/3 to 2009/10	Bone	Letrozole	SD	TTP = 7 months
4th	2009/10 to 2010/10	Bone,lung	Goserelin + anastrozole	SD	TTP = 12 months
5th	2010/10 to 2011/9	Bone,lung	Goserelin + exemestane	SD	TTP = 11 months
6th	2011/9 to 2012/7	Bone,lung	Medroxyprogesterone	SD	TTP = 10 months
7th	2012/8 to 2013/2	Bone,lung	Goserelin + fulvestrant	SD	6^+^ months

*SD* stable disease, *TTP* time to progression.

## Discussion

Male breast cancer is rare, accounting for 1% of all breast cancer, and is also rare in male malignant tumors. Endocrine treatments are important in hormone-receptor-positive male breast cancer. Tamoxifen remains the gold-standard adjuvant treatment. Anastrozole, letrozolea and exemestane as AI can inhibit the action of the enzyme aromatase, which converts androgens into estrogens by a process called aromatization, therefore are used in the treatment of breast cancer. Goserelin is an injectable gonadotropin-releasing hormone superagonist, also known as a luteinizing hormone-releasing hormone (LHRH) agonist, and is used to suppress production of the sex hormones (testosterone and estrogen). For male breast cancer, goserelin,which suppresses production of testosterone, may be better for the effect of AI. Medroxyprogesterone is a potent full agonist of the AR(androgen receptor) by hypothalamus pituitary adrenal axis suppression. Its activation of the AR has been shown to play an important and major role in its antigonadotropic effects and in its beneficial effects against breast cancer. This patient was treated with goserelin plus AI and maintained long-term clinical benefit. To date there has been little clinical study onthe subsequent treatment after disease progression following AI treatment for male breast cancer.

Fulvestrant is novel steroidal ER antagonist lacking agonist effects. By covalent binding to the ER, receptors are rapidly downregulated, resulting in a decrease of cellular ER levels and complete abrogation of estrogen-sensitive gene transcription [1].In postmenopausal women, fulvestrant has been shown to be effective and safe in patients in whom tamoxifen or AI have failed [2]. At present, some clinical studies of fulvestrant used in male breast cancer have been reported [3]. However, endocrine treatment with Goserelin plus fulvestrant for male breast cancer has not been reported in the literature so far. Our patient is a 44-year-old man, so we thought Goserelin,which suppresses production of testosterone may be beneficial with fulvestrant. He has hormone-dependent breast cancer, and sustainable endocrine treatments have achieved long-lasting clinical benefit (3 years).

Since 2009, the patient was under easy endocrine treatments, with good tolerability, avoiding adverse effects of chemotherapy, and improved quality of life. He had more time with his family and work in society.Our oncologist proposes that the treatment for advanced breast cancer should prolong survival, maintain quality of life, and make the patient happy.

## Conclusion

For male hormone-positive breast cancer goserelin plus AI or fulvestrant maybe good choices, especially the patient is hormone-sensitive.

## Consent

Written informed consent was obtained from the patient for publication of this case report.

## Abbreviations

AI: aromatase inhibitors
ER: estrogen receptor
PrR: progesterone receptor.
